# Mapping metabolic dependences and capacities using ATP as a biomarker

**DOI:** 10.21203/rs.3.rs-4836421/v2

**Published:** 2025-04-23

**Authors:** Jose Marin, Amanda Fuchs, Tatiana Tarasenko, Emily Warren, Martha Kirby, Stacie Anderson, Eliza M. Gordon-Lipkin, Shannon Kruk, A. Phillip West, Peter J. McGuire

**Affiliations:** 1. Metabolism, Infection and Immunity Section, National Human Genome Research Institute, National Institutes of Health, Bethesda, MD; 2. Flow Cytometry Core, National Human Genome Research Institute, National Institutes of Health, Bethesda, MD; 3. The Jackson Laboratory, Bar Harbor, ME

**Keywords:** Bioenergetics, MitE-Flo (Mitochondrial/Energy Flow Cytometry), ATP, immunometabolism, influenza

## Abstract

Metabolic dependences and capacities highlight a cell’s reliance on specific pathways to meet its bioenergetic needs, with these pathways being interrogated using chemical inhibitors to assess their significance. While surrogate markers of bioenergetics (e.g., oxygen consumption, protein translation inhibition) have yielded important insights, we asked whether ATP could be used as a biomarker. To address this gap, we developed Mitochondrial/Energy Flow Cytometry (MitE-Flo), a method that evaluates the contributions of glycolysis, fatty acid/amino acid oxidation (FAO/AAO), and oxidative phosphorylation (OXPHOS) to cellular ATP content. In models of mitochondrial disease due to complex I or complex IV deficiency, we identified impaired OXPHOS with a compensatory shift to glycolysis. To define the utility of ATP monitoring in immune niches, we analyzed lungs from mice infected with influenza. CD4^+^ and CD8^+^ T cells, as well as macrophages, demonstrated a shift from OXPHOS dependence to glycolysis dependence during infection, consistent with activation. Using ATP as a biomarker to define metabolic dependences and capacities provides valuable insights into disease states and mixed cell populations, thereby enhancing the metabolism research toolkit.

## INTRODUCTION

Metabolic dependences and capacities underscore the reliance on specific pathways by cells to fulfill their bioenergetic requirements. Interrogation of these parameters involves employing chemical inhibitors to evaluate the importance of select pathways. Over the past decade, technological advancements in high-resolution respirometry (i.e., Oroboros), extracellular flux analysis (i.e., Seahorse), and Single Cell ENergetIc metabolism by profilIng Translation inhibition (SCENITH) [1], have enhanced our capabilities for profiling glycolysis, fatty acid oxidation (FAO) and oxidative phosphorylation (OXPHOS) [2]. Although these technologies provide an informative surrogate metric of bioenergetics, they oftentimes do not directly correlate with ATP production [3]. Advancing the field of metabolism necessitates an additional platform that determines metabolic dependences by focusing on ATP as a biomarker.

Based on this need, we asked whether ATP could be used as a readout for determining metabolic dependences and capacities. To address this question, we developed Mitochondrial/Energy Flow Cytometry (MitE-Flo), a method that leverages a fluorescent ATP sensor with targeted inhibitors and the precision of flow cytometry. MitE-Flo enables bioenergetic profiling of disease states, such as mitochondrial disease, and immune niches during infection, providing unprecedented insights. By quantifying ATP directly and profiling cellular identities and activation states, MitE-Flo offers a complementary tool for the comprehensive evaluation of bioenergetics.

## RESULTS

### Cells with distinct bioenergetic capacities display varying ATP levels

**Figure 1.**
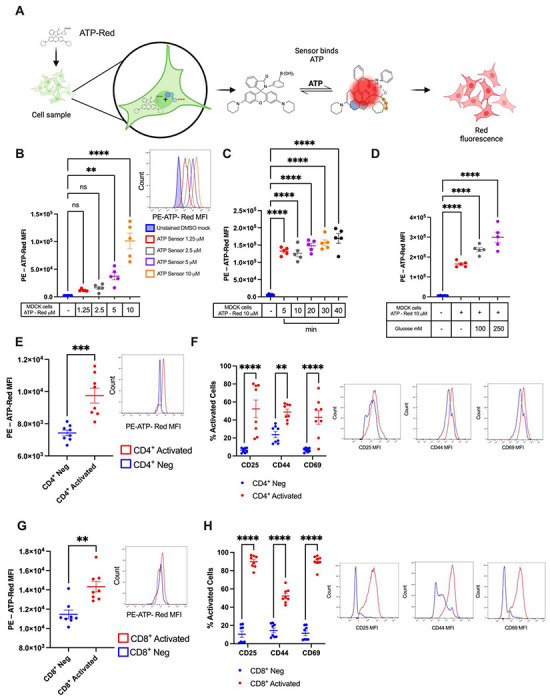
ATP-Red responds to changes in cellular ATP content. A: ATP-Red binding mechanism in living cells. B: Graph of increasing concentration of the ATP-Red on MDCK cells stained for 20 minutes incubated at 37 C and 5% CO_2_ with Optimem medium without fetal bovine serum, a histogram of the different concentrations tested in MDCK cells by flow cytometry (N=5) is shown. C: Graph of increasing kinetics of the 10 M ATP-Red on MDCK cells stained from 5 to 40 minutes incubated at 37 C CO_2_ with Optimem medium without fetal bovine serum (N=5). D: Induction of ATP production in MDCK cells by adding glucose in increasing concentrations from 100 mM to 250 mM stained with ATP-Red 10 M for 20 minutes at 37 C and 5% CO_2_ (N=4). E-H: Shift of the 10 M ATP-Red in MDCK, CD4^+^ and CD8^+^ activate or no cells from murine spleens, stained cells for 20 minutes at 37 C and 5% CO_2_ (N=5). A-C: Ordinary one-way ANOVA by Multiple Comparison Test: *p < 0.05; **** p < 0.0001. E-H: Mann-Whitney test *p < 0.05; **** p < 0.0001.

Cells that are metabolically active generally exhibit higher levels of ATP due to their increased bioenergetic demands. The terminal phosphate group of ATP carries a highly negative charge which can be mobilized via various chemical reactions within the cell, providing energy that allows cellular work to be performed [4]. To profile ATP production in cells with varying bioenergetic capacities, we utilized ATP-Red, a fluorescent probe that specifically targets ATP, shows a strong positive correlation with ATP concentration, and does not cross-react with other metabolites [5]. In the absence of ATP, the probe forms a closed ring structure. When ATP is present, the covalent bonds between boron and ribose are broken by the negative charge, opening the ring, producing fluorescence (Figure 1A). Based on these properties, we hypothesized that profiling differences in cellular ATP content could help determine metabolic profiles by flow cytometry. We began by evaluating the optimal concentration of ATP-Red for flow cytometry by serial dilution (1.25 M to 1.10 M) in Madin-Darby canine kidney cells (MDCK cells, 0.2x10^6^ cells/well). A dose response curve was observed where higher concentrations of ATP-Red (5 and 10 M) correlated with stronger fluorescence intensities (Figure 1B). Cell viability was unaffected by ATP-Red concentration (Figure S1A). Next, we sought to determine the optimal timing for incubation. We observed that all timepoints studied displayed significantly robust fluorescence intensities relative to control (Figure 1C); however, timepoints greater than 20 minutes affected cell viability (Figure S1B). Based on our results, we chose the following conditions for subsequent experiments: ATP-Red (10 M) incubated for 20 minutes.

We next asked whether our experimental conditions could detect differences in cellular ATP content between cell lines with differing metabolic activity. Compared to unlabeled cells, we observed a significant shift in ATP-Red fluorescence in MDCK cells (canine kidney cells, (Figure S1C)), unstimulated splenic CD4^+^ and CD8^+^ T cells (murine, Figures S1D–E) and lymphoblastoid cell lines human, (Figure S1F) to varying degrees. For ATP-Red to be useful in profiling metabolic dependences in bioenergetics, it must be able to respond to changes in cellular metabolism, i.e., fluctuations in ATP concentration. To answer this question, we first applied glucose at increasing concentrations 30 minutes before staining with ATP-Red. We observed that glucose supplementation (100 mM and 250 mM) augmented ATP production (Figure 1D) with normal viability (Figure S1G).

After demonstrating ATP concentrations in supplemented and deficiency states, we next assessed the dynamics of ATP production in activated cells. T cells undergo metabolic reprogramming following activation which includes a marked increase in glycolysis, OXPHOS, and ATP production [6]. To examine changes in ATP content due to cellular activation, we stimulated mouse splenic T cells with anti-CD3/CD28 for 72 hours. Flow cytometric analysis with ATP-Red displayed increases in ATP concentration of CD4^+^ (Figure 1E) and CD8^+^ (Figure 1G) T cells along with markers of activation (Figures 1F and 1H).

### Differential glycolytic dependence revealed through ATP

**Figure 2.**
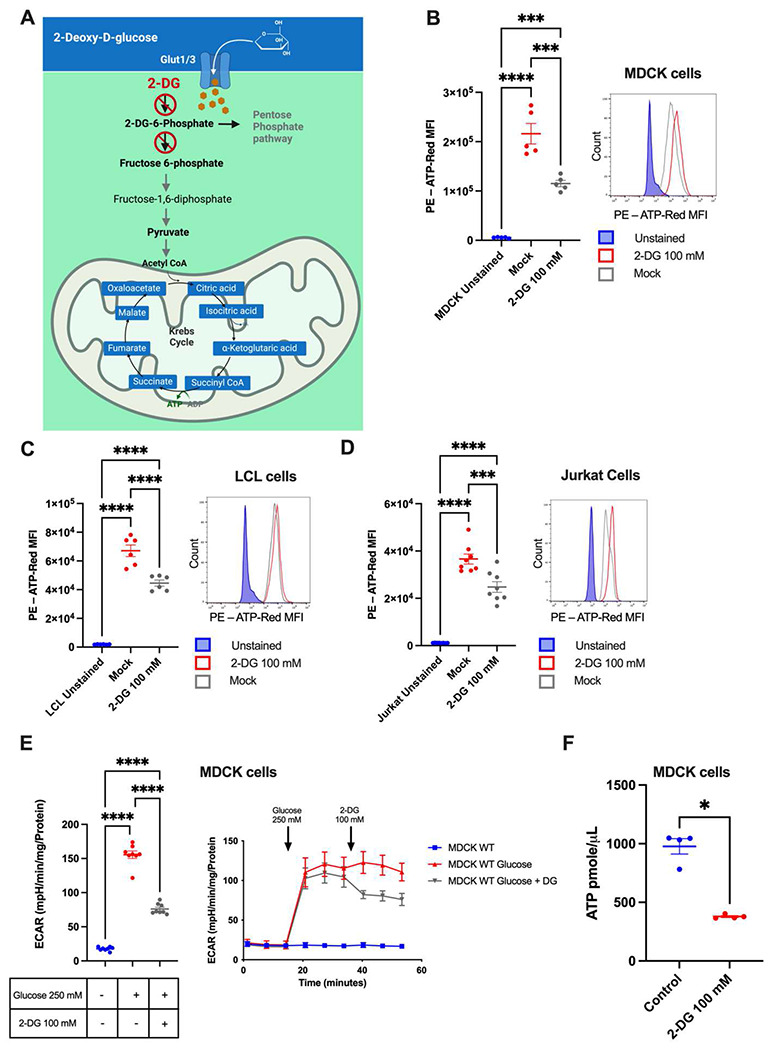
Profiling ATP production from glycolysis with ATP-Red. A: The effect of 2-DG on glycolysis. B: MDCK cells were treated with 100 mM 2-DG for 30 minutes and stained with 10 M ATP-Red for 20 minutes at 37 C and 5% CO_2_, flow cytometry histogram is shown (N=5). C: LCL cells were treated with 100 mM 2-DG for 30 minutes and stained with 10 M ATP-Red for 20 minutes at 37 C and 5% CO_2_, flow cytometry histogram is shown (N=5). D: Jurkat cells were treated with 100 mM 2-DG for 30 minutes and stained with 10 M ATP-Red for 20 minutes at 37 C and 5% CO_2_, flow cytometry histogram is shown (N=5). E: Measurement of extracellular acidification rate (ECAR) in MDCK cells (4x10^5^ cells/well) results of 8 replicates are shown. F: MDCK cells were treated with 100 mM 2-DG for 30 minutes and later the ATP concentration was measured by colorimetric method (N=5). B-C: Ordinary one-way ANOVA by Multiple Comparison Test: *p < 0.05; **** p < 0.0001. E-F: Mann-Whitney U *p < 0.05.

As a major energy generating pathway in intermediary metabolism, glycolysis has evolved in nearly all organisms, with glucose being the most common source of cellular energy and a substrate for many biochemical processes [7, 8]. By inhibiting hexokinase and glucose-6-phosphate isomerase, 2-deoxyglucose (2-DG) is an important tool for studying substrate level phosphorylation and glucose oxidation (Figure 2A) [9]. To determine the utility of ATP-Red in defining ATP production from glycolysis (i.e., glycolytic dependence), we treated MDCK cells, Jurkat cells, and lymphoblastoid cell lines (LCLs) with 2-DG. These cells were chosen due to their distinct origins, roles, and metabolic demands. To ascertain the optimal concentration of 2-DG for our experiments, we evaluated cell viability across a gradient of doses. The concentration of 100 mM was identified as the ideal dosage, striking a balance between effective inhibition and maintaining cell viability (Figures S2A–S2F). Following 2-DG treatment, MDCK cells (0.2x10^6^ cells/well) showed a decrease in ATP fluorescence, indicating a glycolytic dependence of 48% (Figure 2B). Similar to MDCK cells, ATP fluorescence in LCLs (0.2x10^6^ cells/well) and Jurkat cells (0.2x10^6^ cells/well) was also decreased (Figure 2C and 2D), albeit to lesser extent, 28% and 27%, respectively.

To corroborate glycolytic suppression by 2-DG, we performed extracellular flux analysis on MDCK cells (0.4x10^4^ cells/well), where we replicated our flow cytometry protocol timing: 250 mM glucose was injected into the well at 20 minutes followed by 100 mM 2-DG 20 minutes later. With this method, MDCK cells experienced a 51% decrease in glycolysis (Figure 2E). Additionally, we determined the ATP concentration with a colorimetric method in MDCK cells treated with 100 mM 2-DG. ATP concentration was reduced by 61% compared to the untreated control (Figure 2F) substantiating our results obtained with ATP-Red (Figures 2B–2D).

### Differential OXPHOS dependence revealed through ATP

**Figure 3.**
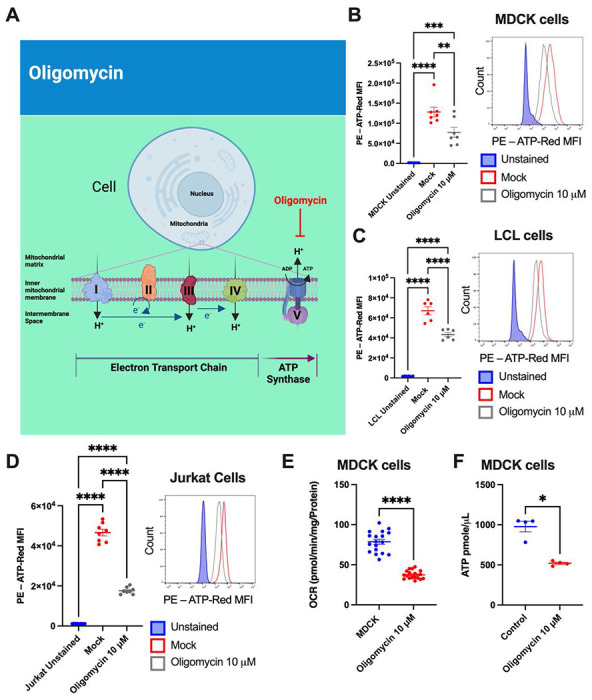
Profiling ATP production from OXPHOS with ATP-Red. A: Inhibitory effect of oligomycin over the ATP synthase complex in OXPHOS. B: MDCK cells were treated with 10 M oligomycin for 30 minutes and stained with 10 M ATP-Red for 20 minutes at 37 C and 5% CO_2_, flow cytometry histogram is shown (N=7). C: LCL cells were treated with 10 M oligomycin for 30 minutes and stained with 10 M ATP-Red for 20 minutes at 37 C and 5% CO_2_, flow cytometry histogram is shown (N=6). D: Jurkat cells were treated with 10 M oligomycin for 30 minutes and stained with 10 M ATP-Red for 20 minutes at 37 C and 5% CO_2_, flow cytometry histogram is shown (N=8). E: Oxygen consumption rate (OCR) of 10 M oligomycin over the ATP synthase complex inhibition assay in MDCK cells (4x10^5^ cells/well, N=18). F: MDCK cells were treated with 10 M oligomycin for 30 minutes and later the ATP concentration was measured by colorimetric method (N=4). B-D: Ordinary one-way ANOVA by Multiple Comparison Test: *p < 0.05; **** p < 0.0001. E-F: Mann-Whitney U *p < 0.05. **** p < 0.0001.

**Figure 4.**
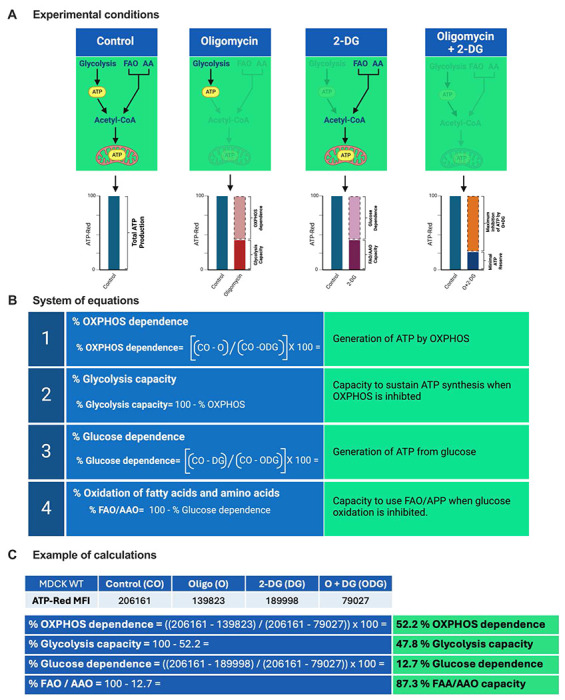
Profiling ATP production with ATP-Red. A: Experimental conditions for the evaluation of the contribution of different metabolic pathways to ATP production in cells under control conditions and inhibitory treatments. In the control condition, ATP production occurs through glycolysis and oxidative phosphorylation (OXPHOS). Inhibition of OXPHOS with oligomycin demonstrates that the measured ATP comes solely from glycolysis. Inhibition of glycolysis with 2-deoxyglucose (2-DG) significantly reduces ATP production, highlighting the dependence on glycolysis. The combination of oligomycin and 2-DG abolishes ATP production, indicating that both pathways are essential. The bar graphs show the relative percentage of ATP produced under each experimental condition. B: Calculation of the relative contributions of different metabolic pathways to ATP production in cells. C: Example of calculations with equations proposed in figure 4B.

Mitochondria are ubiquitous organelles within eukaryotic cells due to their essential function of supplying cellular energy in the form of ATP [10]. Originally identified as an antibiotic produced by fungi, oligomycin is a potent inhibitor of mitochondrial ATP synthase (Figure 4A), and useful in assessing OXPHOS dependence [11]. To profile OXPHOS dependence with ATP-Red, we treated various cell lines with oligomycin. Optimal dosing of oligomycin (10 M) was determined as above (Figure S3A and S3F). We first evaluated the effect of oligomycin on ATP production in MDCK cells (0.2x10^6^ cells per condition). With oligomycin, we observed a reduction in ATP production (Figure 3B), suggesting and OXPHOS dependence of 39%. In LCL cells (0.2x10^6^ cells/well) and Jurkat cells (0.2x10^6^ cells/well), oligomycin (10 M) also decreased ATP fluorescence (Figure 3C and 3D), indicating OXPHOS dependences of 35% and 43%, respectively. To corroborate our findings, we measured the OCR by extracellular flux analysis in MDCK cells (0.4x10^4^ cells/well). Following oligomycin treatment, OCR fell by 52% (Figure 3E). Additionally, we determined the ATP concentration with a colorimetric method in MDCK cells treated with oligomycin (10 M). Similar to our ATP-Red results, we observed a reduction in ATP concentration, indicating an OXHOS dependence of 46% (Figure 3F).

### Integration of metabolic dependences for comprehensive bioenergetic profiling (MitE-Flo)

To advance our methodology and establish comprehensive bioenergetic profiles, we incorporated data from selective inhibitors targeting key steps in glycolysis and OXPHOS (Figure 4A), referred to as MitE-Flo. The application of oligomycin provides two critical parameters: OXPHOS dependence, defined as the decrease in fluorescence, and glycolytic capacity, represented by the remaining fluorescence. OXPHOS dependence reflects ATP production derived from the oxidation of glucose, fatty acids, and amino acids, whereas glycolytic capacity signifies metabolic adaptation through substrate-level phosphorylation in response to OXPHOS inhibition. Similarly, treatment with 2-DG quantifies glucose dependence, observed as a decrease in fluorescence. The residual fluorescence following 2-DG treatment corresponds to the FAO and amino acid oxidation (FAO/AAO) capacity, which reflects the mitochondrial ability to oxidize fatty acids and amino acids for ATP synthesis. The combined application of oligomycin and 2-DG enables the determination of minimal ATP reserves, offering a holistic perspective on cellular bioenergetics. These fluorescence changes, measured via flow cytometry, are converted from geometric mean fluorescence intensity (MFI) into percentage values for detailed analysis (Figure 4B). For example, in MDCK cells, OXPHOS and glucose dependences were quantified as 52.2% and 12.7%, respectively. Metabolic adaptations to these perturbations, represented by glycolytic capacity and FAO/AAO capacity, were 47.8% and 87.3%, respectively.

### MitE-Flo delineates metabolic dependences in mitochondrial disease

**Figure 5.**
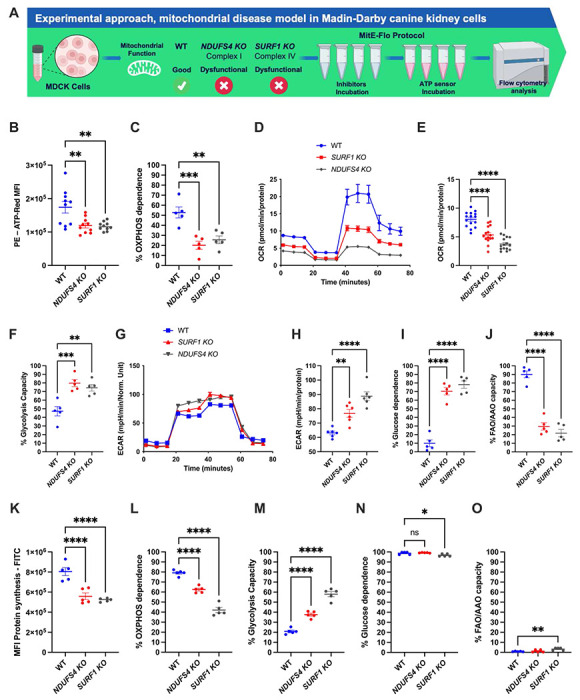
Experimental model of mitochondrial dysfunction in Madin-Darby canine kidney (MDCK) cells. A: Evaluation of mitochondrial function in MDCK Wild Type, *NDUFS4 KO* and *SURF1 KO* cells. B: Relative ATP production under basal conditions is depicted, ATP content in MDCK WT, *NDUFS4 KO* and *SURF1 KO* cells stained with 10 M ATP-Red for 20 minutes at 37 C and 5% CO_2_ (N=10) C: metabolic profile of MDCK WT *NDUFS4 KO* and *SURF1 KO* cells, showing the OXPHOS dependence (%) (N=5). D: Complete sequence of Mito stress experiment is shown for obtain the oxygen consumption values in different timepoints in MDCK WT *NDUFS4 KO* and *SURF1 KO* cells, analyzed with the Seahorse extracellular flux analyzer. E: Basal respiration in cells MDCK WT *NDUFS4 KO* and *SURF1 KO*, three individual experiments with 7 replicates each (N=3). F: Comparison of glycolytic capacity (%), in cells MDCK WT *NDUFS4 KO* and *SURF1 KO.* G: Complete sequence of Glycolysis stress experiment is shown for obtain the extracellular acidification rate values in different timepoints in MDCK WT *NDUFS4 KO* and *SURF1 KO* cells, analyzed with the Seahorse extracellular flux analyzer. H: Extracellular acidification after oligomycin injection to determine glycolytic capacity in cells MDCK WT *NDUFS4 KO* and *SURF1 KO*, three individual experiments with 6 replicates each (N=3). I: glucose dependence (%) in cells MDCK WT *NDUFS4 KO* and *SURF1 KO* (N=5). J: Fatty acid and amino acid oxidation capacity (%) in cells MDCK WT *NDUFS4 KO* and *SURF1 KO*, determined as the proportion of ATP generated from these metabolic pathways when glucose oxidation is inhibited (N=5). K: The relative activity of protein synthesis under basal conditions is represented. Protein synthesis in MDCK WT, NDUFS4 KO and SURF1 KO cells was determined using Click-iT^™^ HPG Alexa Fluor^™^ 488 Protein Synthesis Assay Kit according to the manufacturer’s recommendations (N=5). L: Metabolic profile of MDCK WT, *NDUFS4 KO*, and *SURF1 KO* cells, showing OXPHOS dependence (%) (N=5). M: Comparison of glycolytic capacity (%) in MDCK WT, *NDUFS4 KO*, and *SURF1 KO* cells. N: Glucose dependence (%) in MDCK WT, *NDUFS4 KO*, and *SURF1 KO* cells (N=5). O: Fatty acid and amino acid oxidation capacity (%) in MDCK WT, *NDUFS4 KO*, and *SURF1 KO* cells, expressed as the proportion of ATP generated from these pathways when glucose oxidation is inhibited (N=5). B-O: Ordinary one-way ANOVA by Multiple Comparison Test: *p < 0.05; **** p < 0.0001.

Cells with mitochondrial dysfunction exhibit metabolic adaptations to mitigate impaired OXPHOS. For instance, in a mouse model of mitochondrial encephalopathy, increased glycolytic flux compensates for compromised OXPHOS functionality [12]. To evaluate the utility of MitE-Flo in characterizing the metabolic phenotype of disease states associated with impaired bioenergetics, we studied cells deficient in either NADH:ubiquinone oxidoreductase subunit S4 (NDUFS4) or Surfeit 1 (SURF1) (Figure 5A). NDUFS4 is a critical subunit of ETC complex I, whereas SURF1 functions as an assembly factor for cytochrome c oxidase (COX, complex IV). Deficiency in either gene results in OXPHOS dysfunction. MDCK cells were selected for targeted CRISPR editing due to their robust bioenergetic profiles established above. As anticipated, NDUFS4 and SURF1 knockout (KO) cells displayed significantly reduced ATP-Red levels, consistent with OXPHOS deficiency (Figure 5B). OXPHOS dependence was approximately 20% lower in NDUFS4 and SURF1 KO cells compared to WT controls (Figure 5C), corroborated by extracellular flux analyses (Figures 5D and 5E). Glycolytic capacity was elevated by 25–30% in these KO cells, as expected (Figure 5F), with corresponding confirmation from extracellular flux measurements (Figures 5G and 5H). Glucose dependence in the mitochondrial disease models was 60–70% higher than in WT cells (Figure 5I), while FAO/AAO capacity was markedly reduced (Figure 5J), consistent with predictions for these metabolic phenotypes. Additionally, we assessed the metabolic profile of MDCK cells using a methodology adapted from Arguello (insert reference), employing the Click-iT Protein Synthesis Assay (Thermo Fisher Scientific) to modulate protein synthesis (Figure 5K). Our results demonstrated an inhibition profile comparable to that observed with the ATP sensor assay (Figure S5A). To further validate the assay, we introduced additional specific inhibitors of protein synthesis (Figure S5B), noting that no cell death occurred at low concentrations of these inhibitors (Figure S5C). The metabolic profile derived from protein synthesis analysis showed strong agreement with that obtained from ATP analysis, particularly for parameters such as % OXPHOS dependence and % glycolytic capacity (Figures 5L–M). However, discrepancies were observed for % glucose dependence and % FAA/AAO (Figures 5N–O). We hypothesize that these differences arise from the time required for cellular protein pools to adapt to low or absent ATP levels. In this context, the MitE-Flo assay, by directly measuring ATP, may provide a more dynamic and accurate representation of these metabolic pathways.

### MitE-Flo delineates metabolic dependences in immune niches

**Figure 6.**
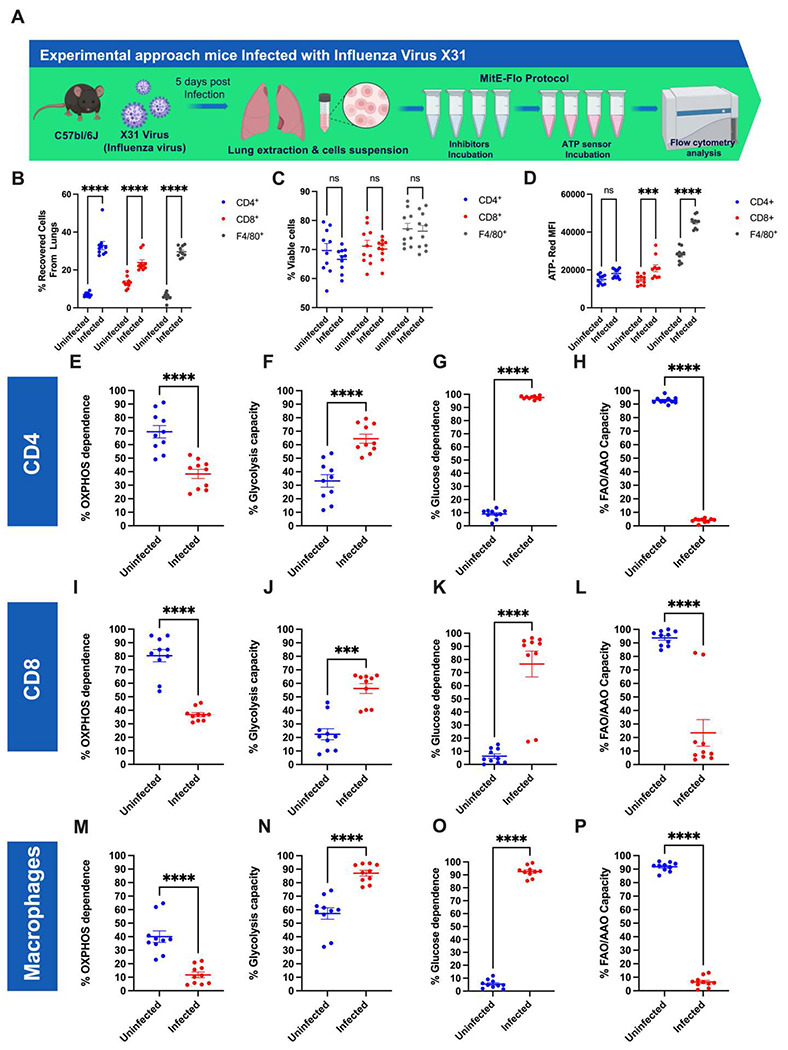
Utility of e^−^Flo in complex cell populations. A: Murine respiratory infection model with influenza virus. B: Percentage of cells recovered in uninfected vs infected lungs, C: (%) Live cell populations selected using Aqua live and dead cell stain are shown, within this population cell subpopulations are selected for, CD4^+^, CD8^+^, and F4/80^+^, in uninfected and infected mice. B: The metabolic profiles obtained for the CD4^+^, CD8^+^, and F4/80^+^, cell populations are shown on uninfected and infected mice. D: Relative ATP production under basal conditions is depicted, ATP content in CD4^+^, CD8^+^, and F4/80^+^ cells stained with 10 M ATP-Red for 20 minutes at 37 C and 5% CO_2_ (N=10) E-P: metabolic profile of CD4^+^, CD8^+^, and F4/80^+^ cells, showing the OXPHOS dependence (%), Comparison of glycolytic capacity (%), glucose dependence (%) and Fatty acid and amino acid oxidation capacity (%) (N=10). B-D: Two-way ANOVA by Multiple Comparison Test: **** p < 0.0001. Mann-Whitney test ***p < 0.05; **** p < 0.0001.

A key advantage of flow cytometry is its capacity to deconvolute complex cell populations. To evaluate the ability of MitE-Flo to generate bioenergetic profiles from heterogeneous cell mixtures, such as those found in immune niches, we isolated cell populations from mouse lungs infected with influenza A virus (IAV, X31 subtype) (Figure 6A). Samples were stained with a panel of antibodies targeting anti-mouse CD4 (CD4^+^ T cells), anti-mouse CD8 (CD8^+^ T cells), and anti-mouse F4/80 (monocytes/macrophages). Leukocyte populations were selected by gating live cells from the lung homogenate (Figure S6). As expected, infected animals yielded higher numbers of cells recovered from the lungs compared to uninfected controls (Figure 6B), with comparable cell viability between the two groups (Figure 6C). Total ATP levels were elevated in CD8^+^ T cells and macrophages when compared to CD4^+^ T cells, reflecting their increased bioenergetic demands (Figure 6D). The bioenergetic profiles of cells from infected lungs were consistent with previous findings in the biomedical literature regarding activated immune cells [13]. Specifically, OXPHOS dependence was reduced across all cell types (Figures 6E, 6I, and 6M), while glucose dependence was markedly increased (Figures 6G, 6K, and 6O). Glycolytic capacity also showed a significant increase in all cell populations (Figures 6C, 6F, and 6J), reflecting metabolic adaptation to immune activation. Conversely, FAO/AAO capacity was suppressed in all analyzed cell types, aligning with known metabolic shifts in activated immune cells.

## DISCUSSION

In this contribution, we have established that ATP can be used as a biomarker to determine metabolic dependences using MitE-Flo, a flow cytometry-based bioenergetics platform. Applying MitE-Flo to normal physiology as well as pathology, we probed the metabolic nuances of models of mitochondrial disease and germinal centers. *NDUFS4* and *SURF1* knockout (KO) cells demonstrated allostatic adaptations in glycolytic dependence secondary to OXPHOS dysfunction. Furthermore, MitE-Flo illuminated the differential metabolic dependences of cells within an immune niche: T cells and macrophages.

The detailed analysis of ATP, a crucial indicator of cellular energy status, is integral for unraveling metabolic processes [14]. Employing ATP-Red for its specificity for ATP fluctuations allows for precise bioenergetic profiling [5]. MitE-Flo leverages this specificity, enabling the direct measurement of intracellular ATP via flow cytometry, and thus facilitates the identification of cellular metabolic phenotypes. This is particularly revealing of the metabolic pathways, glycolysis, FAO, and OXPHOS, that are engaged to meet the bioenergetic demands, especially during the metabolic shifts from OXPHOS to glycolysis upon cell activation [30].

Insights into ATP dynamics are foundational for elucidating the concept of metabolic allostasis [15], a critical cellular process for maintaining energy balance that is necessary for cellular viability and stress response adaptation. It is in this pursuit that the concept of metabolic dependence is highlighted, where cells exhibit a preference for certain pathways to generate ATP. Utilizing metabolic inhibitors, we can discern these metabolic preferences, providing insight into how cells strive to maintain energy balance. In the context of our *NDUFS4* and *SURF1* KO cells, an increase in glycolytic dependence in response to OXPHOS deficiency indicates a shift within metabolic allostasis, revealing an adaptive strategy rather than a state of metabolic inflexibility. This observation hints at broader systemic changes that, under prolonged or excessive mitochondrial stress, could potentially lead to either a resilient or an enduringly altered metabolic phenotype [15–17].

The glycolytic, FAO/AAO, and OXPHOS pathways contribute variably to cellular ATP production, shaped by the cell’s energy demands and specific context. Enhanced energy requirements typically prioritize glycolysis for its swift ATP yield, while FAO is more integral during stable energy conditions [18]. Despite the role of OXPHOS as a primary ATP source, certain diseases and activation states in cells, such as T cells, can shift the preference towards glycolytic dependence, even when oxygen is plentiful [14, 19]. The interplay among these metabolic pathways reflects a cell’s adaptability (i.e., allostasis), influenced by factors like energy needs, oxygen availability, and cell status; attributes that are also reflected in the metabolic nuances of cells with immune niches.

The development of MitE-Flo for metabolic phenotyping represents a significant advancement in the field of cellular metabolism, providing a refined lens for examining the nuances of cellular bioenergetics. Its capacity to assess glycolytic, FAO and OXPHOS/ETC dependences offers a powerful method for characterizing metabolic states, which could be transformative in disease staging. In inflammatory diseases or cancer, where metabolic shifts often correlate with disease activity, MitE-Flo could be utilized to monitor these changes, potentially serving as a biomarker for disease progression or response to therapy [20–24]. The precise data gathered by MitE-Flo on mitochondrial health and function could also aid in diagnostics for a range of diseases characterized by altered bioenergetics, including mitochondrial diseases. Further research and validation are required to confirm its efficacy and reliability in these contexts, which could pave the way for more targeted and effective treatments, informed by detailed bioeneregtic profiles.

In summary, MitE-Flo combines bioenergetic profiling with the granularity of flow cytometry. This platform is robust and allows for the study of disease states and rare cell populations with minimal disturbance. The protocol is quick, inexpensive, and widely accessible due to the availability of reagents and ubiquitous nature of flow cytometry at most research institutions. Most importantly, MitE-Flo selectively and directly measures ATP, the primary energy currency of the cell and biomarker of cellular bioenergetic status.

## MATERIALS AND METHODS

### Cell culture: Madin Darby canine kidney cells (MDCK)

Cells were routinely subcultured every 3 days (72 hours ± 6 hours) at a seeding density of 1x10^5^ cells/cm^2^ or 5x10^4^ cells/cm^2^ (DMEM + 10% FBS + Penicillin-Streptomycin (100 U/mL) in 75 cm^2^ vent-cap T-flasks. The total volume of media was 20 mL per flask. Cultures were maintained at 37 C ± 1 C in a humidified incubator with 5% CO_2_. Cell counts were determined by standard counting technique using an automatic cell counter (1:2 dilution in 0.04% trypan blue). All cultures were maintained using aseptic technique. At the end of the culture, the cells were detached with 2.0 to 3.0 mL of trypsin-EDTA 0.05% solution and incubated for 10 minutes at 37 C ± 1 C in a humidified incubator with 5% CO_2_. The cell suspension was washed with 1X PBS and centrifuged at 1250 RPM for 5 minutes. The cell pellet was resuspended in Optimem media where they were counted using an automatic cell counter (1:2 dilution in 0.04% trypan blue). A concentration of 0.2x10^6^ cells/tube was used for the respective analyses and drug treatments.

### Cell culture: Immortalized line of human T lymphocyte cells (Jurkat)

Cells were routinely subcultured every 2 and 3 days (48 −72 hours). Cultures were maintained at a cell concentration between 1x10^5^ and 1x10^6^ viable cells/mL (RPMI-1640 Medium + 10% FBS + Penicillin-Streptomycin (100 U/mL)) in 25 cm^2^ or 75 cm^2^ culture flasks. Cultures were maintained at 37 C ± 1 C in a humidified incubator with 5% CO_2_. Cell counts were determined by standard counting technique using an automatic cell counter (1:2 dilution in 0.04% trypan blue). All cultures were maintained using aseptic technique. At the end of the culture, the cells were collected, and the cell suspension was washed with 1X PBS and centrifuged at 1250 RPM for 5 minutes, the cell pellet was resuspended in Optimem media where they were counted using an automatic cell counter (1:2 dilution in 0.04% trypan blue). A concentration of 0.2x10^6^ cells/tube was used for the respective analyses.

### Cell culture: Lymphoblastoid cell lines (LCL)

Cells were routinely subcultured every 3 days (72 hours). Cultures were maintained at a cell concentration between 1x10^5^ and 1x10^6^ viable cells/mL (RPMI-1640 Medium + 10% FBS + sodium pyruvate 1 mM + Glutamine 2mM + MEM NEAA 1x + Penicillin-Streptomycin (100 U/mL)) in 25 cm^2^ or a 75 cm^2^ culture flasks. Cultures were maintained at 37 C ± 1 C in a humidified incubator with 5% CO_2_. Cell counts were determined by standard counting technique using an automatic cell counter (1:2 dilution in 0.04% trypan blue). All cultures were maintained using aseptic technique. At the end of culture, the cells were collected, and the cell suspension was washed with 1X PBS and centrifuged at 1000 RPM for 5 minutes, the cell pellet was resuspended in Optimem where they were counted using an automatic cell counter (1:2 dilution in 0.04% trypan blue). A concentration of 0.2x10^6^ cells/tube was used for the respective analyses.

### Measurement of cell respiration with Seahorse flux analyzer

Extracellular flux analysis (Seahorse, Agilent, Santa Clara, CA.) can determine the rate of mitochondrial oxidative phosphorylation, through measurement of the oxygen consumption rate (OCR) measured in (pmol/min/mg/protein), in a 96 microplate-based assay platform. For this methodology, basal OCR was performed on MDCK cells (4x10^5^ cells/well) and obtained by the OCR of the last reading before adding the Oligomycin 10 M. The assay was performed in XF Assay Modified DMEM. Three consecutive measurements were performed under basal conditions and after the sequential addition of the following ATP synthase inhibitor.

### Measurement of extra cellular acidification rate (ECAR) with Seahorse flux analyzer

The extracellular acidification rate (ECAR) was determined in MDCK cells (4x10^5^ cells/well) suing extracellular flux analysis as above and measured in (mpH/min/mg/protein). This was obtained by taking the ECAR of the last reading before adding 250 mM glucose as an inducer of ATP production and increase in extracellular acidification. ECAR was then taken in the last measurement after the addition of 2-DG 100 mM to obtain the inhibitory effect of the drug. The assay was performed in XF Assay Modified DMEM. Three consecutive measurements were performed under basal conditions and after the sequential addition of the following electron transport chain inhibitors.

### Mitochondrial Stress Test Assay

The mitochondrial stress test was performed using the Seahorse XF Cell Mito Stress Test Kit (Agilent Technologies) according to the manufacturer’s protocol. MDCK cells were seeded at a density of 40,000 cells per well in a Seahorse XF96 cell culture microplate and allowed to adhere overnight in DMEM supplemented with 10% fetal bovine serum (FBS) at 37°C and 5% CO_2_. The following day, the cells were washed with pre-warmed Seahorse The assay was conducted by sequentially injecting the following compounds at the specified final concentrations: 1 μM oligomycin to inhibit ATP synthase, 1.5 μM FCCP to uncouple mitochondrial respiration, and a combination of 0.5 μM rotenone and antimycin A to block the electron transport chain. Oxygen consumption rate (OCR) was measured using the Seahorse XF Analyzer, and parameters such as basal respiration, ATP production-linked respiration, maximal respiration, and spare respiratory capacity were calculated. Data were normalized to cell number or protein content determined post-assay using the pierce BCA protein assay kit thermo scientific 23227. All experiments were performed in triplicate and repeated at least three times for reproducibility.

### Glycolytic Stress Test Assay

The glycolytic stress test was conducted using the Seahorse XF Glycolysis Stress Test Kit (Agilent Technologies) following the manufacturer’s protocol. MDCK cells were seeded at a density of 40,000 cells per well in a Seahorse XF96 cell culture microplate and incubated overnight in DMEM supplemented with 10% fetal bovine serum (FBS) at 37°C and 5% CO_2_. On the day of the assay, the cells were washed and incubated in Seahorse XF Assay Medium (without glucose, pyruvate, or glutamine) supplemented with 2 mM glutamine. The plate was equilibrated for 1 hour in a CO_2_-free incubator at 37°C. During the assay, the following compounds were sequentially injected at their final concentrations: 10 mM glucose to initiate glycolysis, 1 μM oligomycin to inhibit mitochondrial ATP production and reveal maximum glycolytic capacity, and 50 mM 2-deoxyglucose (2-DG) to inhibit glycolysis and determine non-glycolytic acidification. The extracellular acidification rate (ECAR) was measured using the Seahorse XF Analyzer. Parameters such as basal glycolysis, glycolytic capacity, glycolytic reserve, and non-glycolytic acidification were calculated. Post-assay normalization was performed using the pierce BCA protein assay kit thermo scientific 23227 assay to account for differences in cell number. All experiments were carried out in triplicate and repeated at least three times to ensure reproducibility.

### Colorimetric ATP

MDCK cells were divided into 4 experimental conditions and treated with the different inhibitory drugs oligomycin (10 M), 100 mM 2-DG and untreated control cells, to which the extraction was performed. of ATP using the recommendations of the kit and the enzymatic reaction was carried out according to the instructions of the kit (ATP kit MAK190 SIGMA), where we later measured the absorbance at a wavelength of 570 nm. We calculated ATP concentrations using a standard curve provided by the manufacturer.

### Drugs stock

The drugs (Oligomycin 5 mM sigma, were resuspended in 100% ethanol, (2-deoxy-D-glucose, 1 M sigma) in Optimem media, for stock generation and were stored in aliquots at −20 °C until use according to the manufacturer’s instructions.

### Metabolic profile by flow cytometry (MitE-Flo)

For the cellular metabolic profile analysis, we used MDCK, LCL, Jurkat, and primary mouse cells at a concentration of 0.2x10^6^ cells/well in Optimem media without fetal bovine serum. The cells were divided into 4 different wells at this same concentration and to which inhibitory drugs were added individually (10 M oligomycin, 100 mM 2-DG), The cells were incubated at 37 C for 30 minutes, then a final 10 M ATP-Red concentration was added plus an LIVE/DEAD^™^ Fixable aqua or LIVE/DEAD^™^ Fixable Near IR (876) Viability Kit probe in a ratio of 1/1000. In the same step cells were labeled with a panel of fluorescent conjugated antibodies targeting specific mouse antigens, including anti-mouse CD4, anti-mouse CD8, anti-mouse in samples from mouse lungs. In the presence of drug treatments, the cells were incubated at 37 C for 20 minutes. After incubation the cells were washed with 1X PBS and centrifuged at 1250 RPM for 5 minutes and resuspended in 1% PFA and subsequently analyzed by flow cytometry.

### Protein synthesis assay

Protein synthesis in MDCK cells was assessed using the Click-iT^™^ HPG Alexa Fluor^™^ 488 Protein Synthesis Assay Kit (Thermo Fisher Scientific) and analyzed by flow cytometry. MDCK cells were seeded at a density of 40,000 cells per well in a 96-well plate and allowed to adhere overnight in DMEM supplemented with 10% fetal bovine serum (FBS) at 37°C in a 5% CO_2_ incubator. The next day, cells were treated with 10 μM oligomycin, 100 mM 2-DG and ODG= 10 μM oligomycin, 100 mM 2-DG inhibitors, then cells were washed with warm phosphate-buffered saline (PBS) and incubated in methionine-free DMEM for 30 min at 37 °C to deplete intracellular methionine stores. Protein synthesis was then quenched by treating cells with 50 μM L-homopropargylglycine (HPG), a methionine analog, for 1–2 h. Specific protein synthesis inhibitors, such as 10 μg/mL cycloheximide, were used as controls to assess inhibition. After labeling, cells were harvested, washed with PBS, and fixed with 4% paraformaldehyde for 15 min at room temperature. Permeabilization was performed using 0.5% Triton X-100 in PBS for 20 min. The Click-iT^™^ reaction was carried out by incubating cells with Alexa Fluor^™^ 488 azide reaction cocktail for 30 min in the dark. After the reaction, cells were washed twice with PBS containing 1% bovine serum albumin (BSA). Fluorescence was analyzed using a CytoFLEX S flow cytometer (Beckman Coulter) equipped with a 488 nm laser for excitation and a 525/40 nm emission filter. A minimum of 20,000 events per sample were acquired, and data analysis was performed using FlowJo software. Mean fluorescence intensity (MFI) values were used to quantify protein synthesis, normalized to unstained controls. All experiments were performed in triplicate and repeated at least three times to ensure reproducibility.

### Data analysis

All values are presented as mean and standard error of the mean (SEM) of a number of independent experiments (>3). As most of our datasets did not display normality, non-parametric tests were applied Mann-Whitney. Comparisons between more than two paired data sets were made using the ordinary one-way ANOVA with a Multiple Comparison Test. For all statistical comparisons we used a Prism 10.2 software, where a p value <0.05 was considered significant. All illustrations were created with BioRender.com.

## Supplementary Material

1

This is a list of supplementary files associated with this preprint. Click to download.

• SupplementaryMaterials.docx

## Data Availability

All data are available in the main text, supplementary materials, or by reasonable request from the authors.

## HIGHLIGHTS

The benefits of MitE-Flo include:

- Commercially available reagents.
- Direct profiling of ATP production from key bioenergetic pathways.
- Ability to distinguish disease states marked by bioenergetic perturbations.
- Metabolic phenotyping of complex cell populations from immune niches.
