# Supplementary material for: Mapping metabolic dependences and capacities using ATP as a biomarker
Source: Res Sq. 2025 Apr 23:rs.3.rs-4836421. Preprint. [Version 2] doi: 10.21203/rs.3.rs-4836421/v2 (PMC12747283; doi:10.21203/rs.3.rs-4836421/v2)
Supplement: 1 [file NIHPPrs4836421v2-supplement-1.pdf]

Supplementary Figures

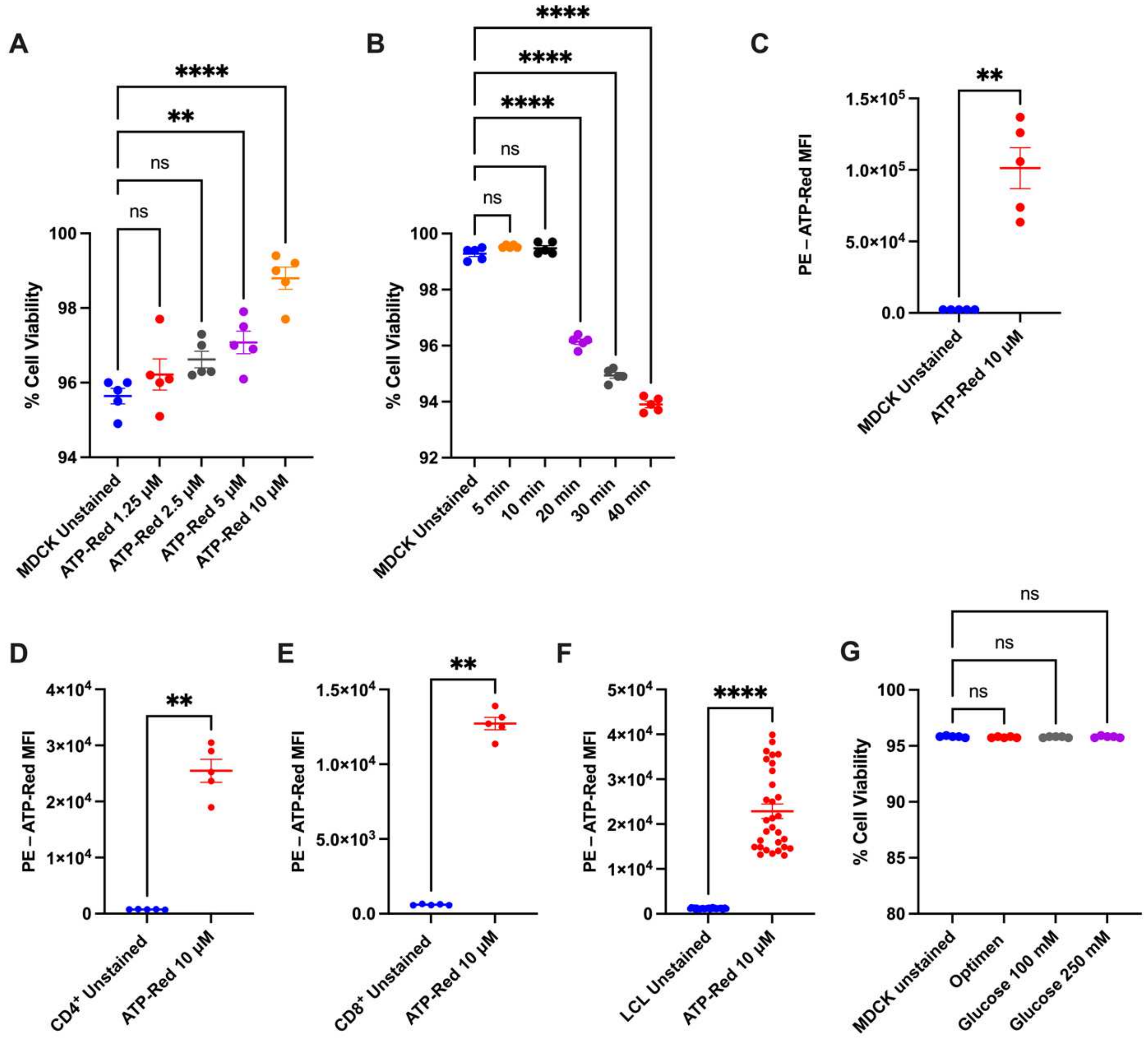

Supplementary Figure 1. A: Percentage of cell viability of MDCK cells treated with different concentrations of ATP-Red for 20 minutes at 37° C and 5% CO_2. B: Percent cell viability of MDCK cells treated in time kinetics with 10 µM ATP-Red for 20 minutes at 37° C and 5% CO_2. C: MDCK cells ATP-Red shift vs unstained cells. D-E: Murine CD4+ and CD8+ cells ATP-Red shift vs unstained cells. F: Human LCL cells ATP-Red shift vs unstained cells. G: Percentage of cell viability of MDCK cells treated with different concentrations of glucose using 10 µM ATP-Red for 20 minutes at 37° C and 5% CO_2. A-B, G: Ordinary one-way ANOVA by Multiple Comparison Test; **** $p < 0.0001$, *$p < 0.05$. C-F: Mann-Whitney test **$p < 0.05$; **** $p < 0.0001$

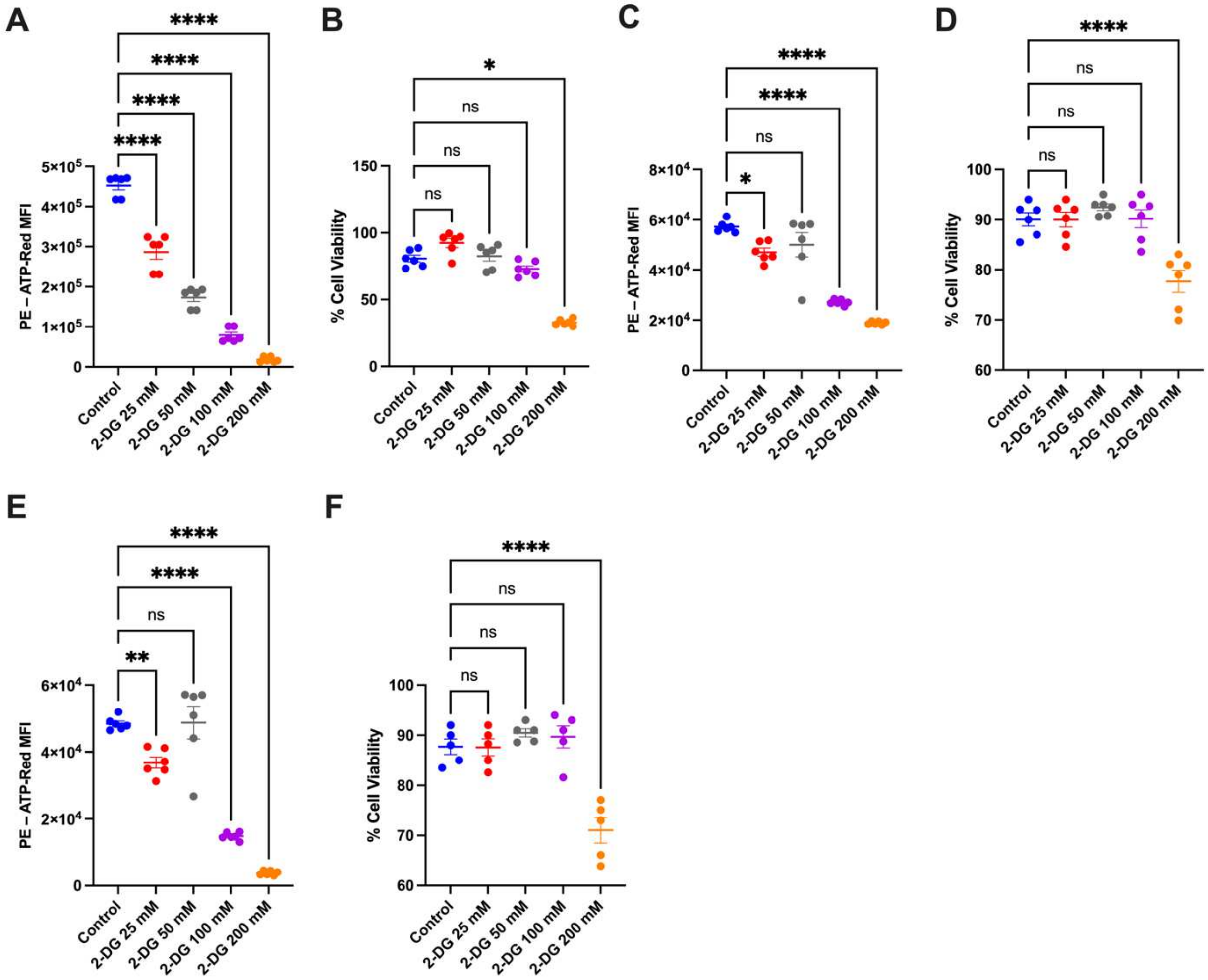

Supplementary Figure 2. A: Fluorescence intensity of MDCK cells treated with different concentrations of 2-DG. B: Percentage of cell viability of MDCK cells treated with different concentrations of 2-DG using 10 M ATP-Red for 20 minutes at 37 C and 5% CO_2. C: Fluorescence intensity of LCL cells treated with different concentrations of 2-DG. D: Percentage of cell viability of LCL cells treated with different concentrations of 2-DG using 10 M ATP-Red for 20 minutes at 37 C and 5% CO_2. E: Fluorescence intensity of Jurkat cells treated with different concentrations of 2-DG. F: Percentage of cell viability of Jurkat cells treated with different concentrations of 2-DG using 10 M ATP-Red for 20 minutes at 37 C and 5% CO_2. A-F: Ordinary one-way ANOVA by Multiple Comparison Test; **** $p < 0.0001$, *$p < 0.05$.

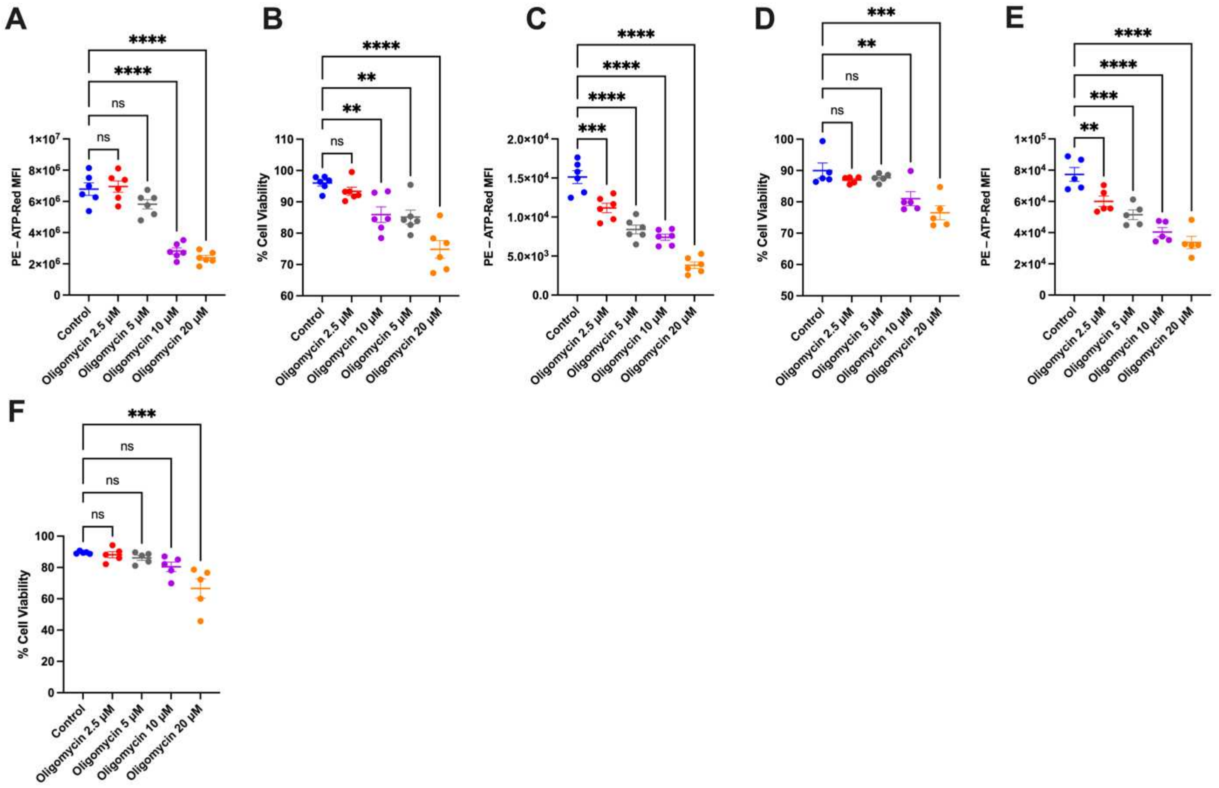

Supplementary Figure 3. A: Fluorescence intensity of MDCK cells treated with different concentrations of oligomycin and 10 μM ATP-Red for 20 minutes at 37° C and 5% CO_2. B: Percentage of cell viability of MDCK cells treated with different concentrations of oligomycin. C: Fluorescence intensity of LCL cells treated with different concentrations of oligomycin and 10 μM ATP-Red for 20 minutes at 37° C and 5% CO_2. D: Percentage of cell viability of LCL cells treated with different concentrations of oligomycin. E: Fluorescence intensity of Jurkat cells treated with different concentrations of oligomycin and 10 μM ATP-Red for 20 minutes at 37° C and 5% CO_2. F: Percentage of cell viability of Jurkat cells treated with different concentrations of oligomycin. A-F: Ordinary one-way ANOVA by Multiple Comparison Test; **** $p < 0.0001$, *$p < 0.05$.

A

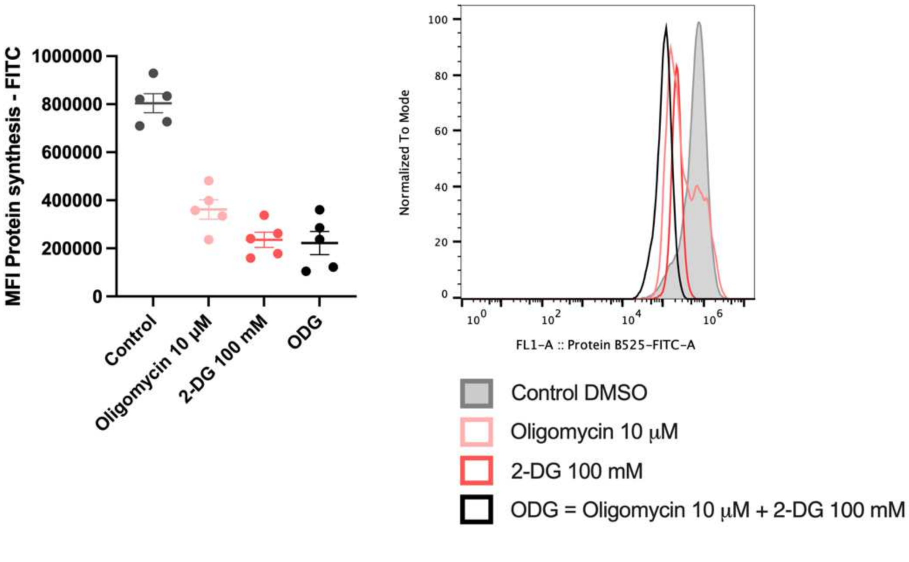
MFI Protein synthesis - FITC
1000000
800000
600000
400000
200000
0
Control
Oligomycin 10 µM
2-DG 100 mM
ODG
Normalized To Mode
FL1-A :: Protein B525-FITC-A
Control DMSO
Oligomycin 10 µM
2-DG 100 mM
ODG = Oligomycin 10 µM + 2-DG 100 mM

B

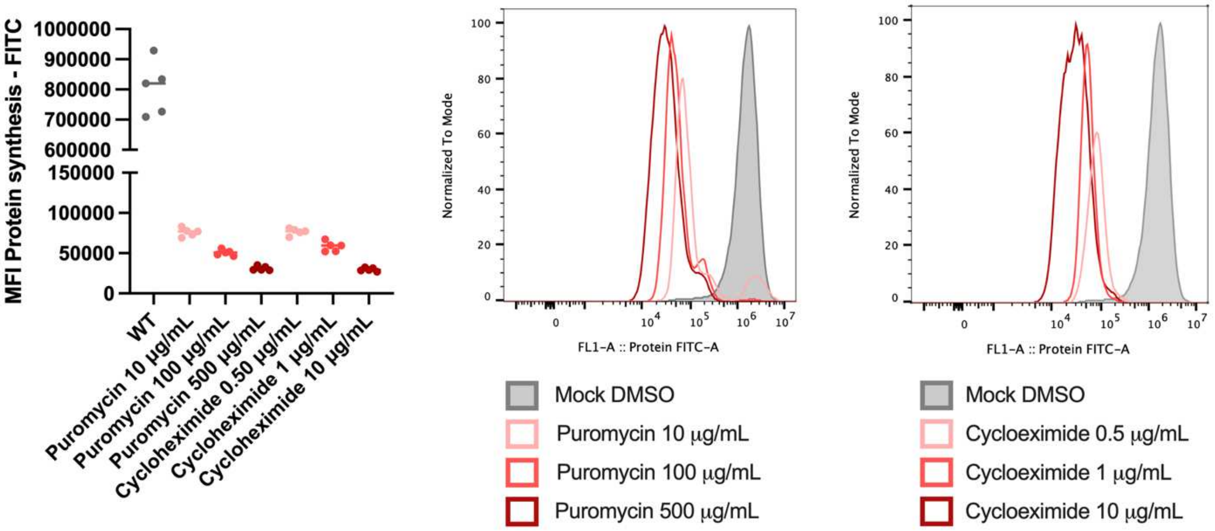
MFI Protein synthesis - FITC
1000000
900000
800000
700000
600000
150000
100000
50000
0
WT
Puromycin 10 µg/mL
Puromycin 100 µg/mL
Puromycin 500 µg/mL
Cycloheximide 0.50 µg/mL
Cycloheximide 1 µg/mL
Cycloheximide 10 µg/mL
Normalized To Mode
FL1-A :: Protein FITC-A
Mock DMSO
Puromycin 10 µg/mL
Puromycin 100 µg/mL
Puromycin 500 µg/mL
Normalized To Mode
FL1-A :: Protein FITC-A
Mock DMSO
Cycloeximide 0.5 µg/mL
Cycloeximide 1 µg/mL
Cycloeximide 10 µg/mL

B

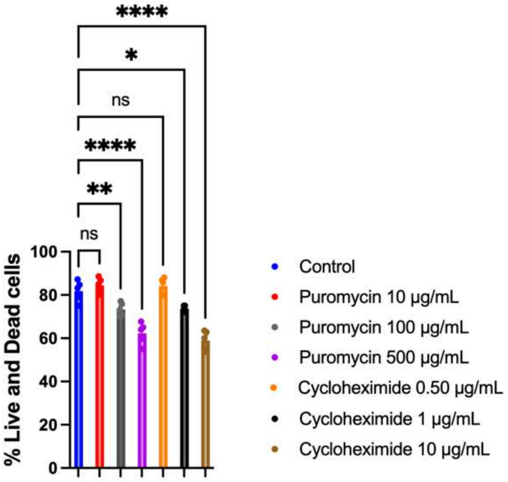
****
*
ns
****
**
ns
% Live and Dead cells
100
80
60
40
20
0
Control
Puromycin 10 µg/mL
Puromycin 100 µg/mL
Puromycin 500 µg/mL
Cycloheximide 0.50 µg/mL
Cycloheximide 1 µg/mL
Cycloheximide 10 µg/mL

Supplementary Figure 5. A: MFI inhibition profile for Click-iT protein synthesis assay in MDCK WT cells with the inhibitors oligomycin 10 µM, 2-DG 100 mM and ODG= oligomycin 10 µM, 2-DG 100 mM. B (left): Click-iT protein synthesis assay in MDCK WT cells in the presence of different concentrations of protein synthesis inhibitors puromycin 10 - 500 µg/mL and cycloheximide 0.5 - 10 µg/mL. B (right): Representative histograms of the fluorescence shift of protein synthesis in the presence of the inhibitors puromycin 10 - 500 µg/mL and cycloheximide 0.5 - 10 µg/mL. C: percentage of viability of control MDCK cells vs inhibitors puromycin 10 - 500 µg/mL and cycloheximide 0.5 - 10 µg/mL.

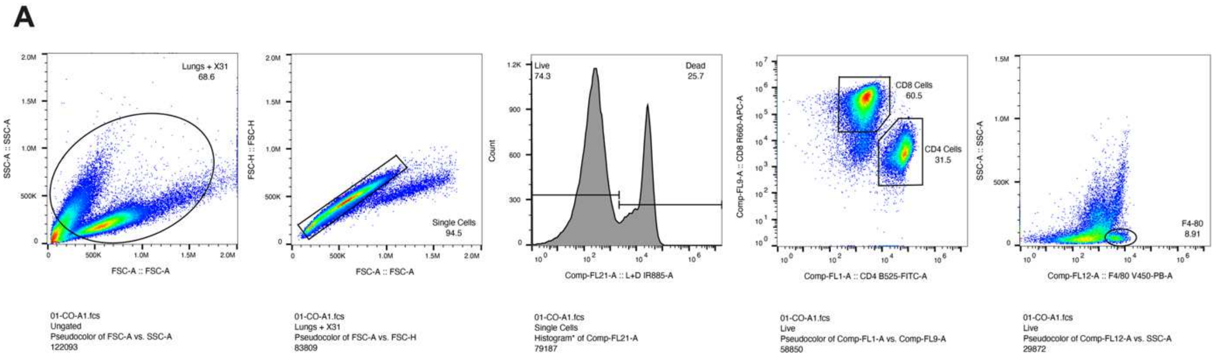

Supplementary Figure 6. A: Example of a gating strategy to select $CD4^+$, $CD8^+$ and $F4/80^+$ macrophage cells in cells from infected or uninfected mice.

Supplementary Files

This is a list of supplementary files associated with this preprint. Click to download.

- SupplementaryMaterials.docx
